# Prof. Paul F. Eve’s Clinical Surgery

**Published:** 1850-11

**Authors:** 


### PROF. PAUL F. EVE'S CLINICAL SURGERY.

We have enjoyed the pleasure of listening to the clinical instruction of Professor Eve, at the Louisville Marine Hospital, and we express but the common opinion of those who have heard him, in saying that his Clinics are at once pleasing, impressive, and highly instructive. We admire, especially, Professor Eve's conservative surgery, which aims at the good of the patient, rather than the gratification of the surgeon; which struggles to lift up the victim of misfortune in preference to exalting the dashing operator. B.

